# Phosphoglycerate mutase 1-mediated dephosphorylation and degradation of Dusp1 disrupt mitochondrial quality control and exacerbate endotoxemia-induced myocardial dysfunction

**DOI:** 10.7150/thno.102647

**Published:** 2024-11-04

**Authors:** Rongjun Zou, Wanting Shi, Mingxian Chen, Miao Zhang, Dan Wu, Haixia Li, Hao Zhou, Yukun Li, Weihui Lu, Chao Li, Xiaoping Fan

**Affiliations:** 1 State Key Laboratory of Traditional Chinese Medicine Syndrome, Guangdong Provincial Hospital of Chinese Medicine, Guangzhou University of Chinese Medicine, the Second Clinical College of Guangzhou University of Chinese Medicine, Guangzhou 510120, Guangdong, China.; 2 Department of Cardiovascular Surgery, Guangdong Provincial Hospital of Chinese Medicine, the Second Affiliated Hospital of Guangzhou University of Chinese Medicine, the Second Clinical College of Guangzhou University of Chinese Medicine, Guangzhou 510120, Guangdong, China.; 3 Guangdong Provincial Key Laboratory of TCM Emergency Research, Guangzhou 510120, Guangdong, China.; 4 Guangzhou Women and Children's Medical Center, Guangzhou Medical University, Guangzhou 510623, Guangdong, China.; 5 Tongde Hospital of Zhejiang Province, No. 234, Gucui road, Hangzhou 310012, China.; 6 Division of Vascular Surgery, the First Affiliated Hospital, Sun Yat-sen University, Guangzhou 510800, China; National-Guangdong Joint Engineering Laboratory for Diagnosis and Treatment of Vascular Disease, First Affiliated Hospital, Sun Yat-sen University, Guangzhou 510080, China.; 7 Xianning Medical College, Hubei University of Science & Technology, Xianning 437000, China.; 8 Department of Cardiology, Beijing Anzhen Hospital, Capital Medical University, Beijing 100029, China.; 9 College of Traditional Chinese Medicine, Shandong University of Traditional Chinese Medicine, Jinan, China.

**Keywords:** Pgam1, Dusp1, LPS, endotoxemia, mitochondrial quality control

## Abstract

**Rationale:** Endotoxemia, caused by lipopolysaccharides, triggers systemic inflammation and myocardial injury by disrupting mitochondrial homeostasis. This study examines the roles of dual specificity phosphatase 1 (Dusp1) and phosphoglycerate mutase family member 1 (Pgam1) in this process.

**Methods:** This study utilized cardiomyocyte-specific *Dusp1* knockout (*Dusp1^Cko^*) and transgenic (*Dusp1^Tg^*) mice, alongside *Pgam1* knockout (*Pgam1^Cko^*) mice, subjected to LPS-induced endotoxemia. Echocardiography was performed to assess cardiac function. Mitochondrial integrity was evaluated using molecular techniques, including qPCR and Seahorse assays. Additionally, molecular docking studies and Western blot analyses were conducted to explore the interaction between Pgam1 and Dusp1.

**Results:** Using single-cell sequencing and human sample databases, Dusp1 emerged as a novel biomarker for endotoxemia-induced myocardial dysfunction. Experiments with cardiomyocyte-specific *Dusp1* knockout (*Dusp1^Cko^*) and *Dusp1* transgenic (*Dusp1^Tg^*) mice showed that *Dusp1* deficiency worsens, while overexpression improves, heart function during LPS-induced myocardial injury. This effect is mediated by regulating inflammation and cardiomyocyte viability. Molecular analyses revealed that LPS exposure leads to Dusp1 dephosphorylation at Ser364, increasing its degradation. Stabilizing Dusp1 phosphorylation enhances mitochondrial function through mitochondrial quality control (MQC), including dynamics, mitophagy, and biogenesis. Functional studies identified Pgam1 as an upstream phosphatase interacting with Dusp1. *Pgam1* ablation reduced LPS-induced cardiomyocyte dysfunction and mitochondrial disorder.

**Conclusions:** Pgam1-mediated dephosphorylation of Dusp1 disrupts mitochondrial quality control, leading to myocardial dysfunction in endotoxemia. Targeting the Pgam1-Dusp1 axis represents a promising therapeutic strategy for improving cardiac outcomes in patients with endotoxemia.

## Introduction

Endotoxemia, a systemic inflammatory state initiated by endotoxins like lipopolysaccharides from Gram-negative bacteria, frequently results in myocardial injury, underlying many sepsis-related cardiac dysfunctions 1, 2. The severity of endotoxemia directly correlates with the degree of cardiac impairment, leading to adverse clinical outcomes 3. Clinical manifestations include biventricular dilation, reduced ejection fraction, and arrhythmias, which can persist in severe cases 3. Despite its clinical significance, the molecular mechanisms underlying endotoxemia-induced cardiac injury remain poorly understood, hindering the development of specific biomarkers and targeted therapies. Further research is crucial to elucidate these mechanisms and translate this knowledge into clinical applications.

Mitochondrial dysfunction, characterized by reduced ATP production and increased ROS generation, is detrimental to cardiac homeostasis. Histopathological examinations of biopsies from septic patients reveal significant mitochondrial structural damage in various organs, including skeletal muscle, heart, liver, and kidneys 4-8. These alterations include outer membrane herniation, vacuole formation, granule accumulation, and matrix edema. Clinically, the severity of mitochondrial dysfunction correlates with sepsis survival rates 9. While these results indicate a key function of for mitochondrial dysfunction in endotoxemia-induced myocardial injury, the upstream signaling events regulating mitochondrial behavior remain to be fully elucidated. These findings implicate mitochondrial dysfunction as a central player in the setting of endotoxemia-induced myocardial dysfunction. However, the primary signaling pathways governing mitochondrial dynamics remain elusive. Previous studies from our laboratory have demonstrated that endotoxemia disrupts myocardial mitochondrial quality control (MQC), leading to increased fission, decreased fusion, impaired mitophagy, and reduced biogenesis 10, 11. This concept is supported by evidence linking MQC dysregulation to exacerbated cardiac dysfunction during endotoxemia. Mechanistically, MQC dysregulation is associated with aberrant Vcp phosphorylation, resulting from the downregulation of Dusp1 expression. While the mechanisms underlying Dusp1 downregulation in the endotoxemic myocardium are unclear, emerging evidence suggests that Dusp1 phosphorylation protects it from proteolytic degradation 12-14. Therefore, it is crucial to investigate whether endotoxemia-induced Dusp1 degradation is mediated by post-transcriptional dephosphorylation.

Recent studies have identified phosphoglycerate mutase family member 1 (Pgam1), a mitochondrial serine/threonine protein phosphatase, as a key regulator of inflammation, expanding its known role in mitochondrial homeostasis 15, 16. Pgam1 deletion in T cells attenuates CD8 and CD4 T cell-related inflammation reaction by enhancing glycolysis and activating mTORC1 and TCR signaling 17. Additionally, Pgam1 deficiency in mice ameliorates helper T cell-dependent inflammation 17. Plasma proteomic analysis in patients with decompensated cirrhosis reveals a positive correlation between Pgam1 and neutrophil activity, oxidative burst, and bilirubin levels 18. In a murine myocardial infarction model, Pgam1 levels are elevated in ischemic hearts, while Pgam1 knockout improves cardiac function and prevents remodeling by suppressing inflammation, apoptosis, and fibrosis 19. Mechanistically, Pgam1's phosphatase activity dephosphorylates substrates crucial for mitochondrial integrity, particularly mitochondrial metabolism 15, thereby modulating oxidative stress and apoptosis induced by mitochondrial dysfunction. This molecular interplay underscores the importance of Pgam1 in fine-tuning inflammatory pathways, with implications for diverse pathologies, including myocardial inflammation and systemic inflammatory disorders. Given the accumulating evidence of Pgam1's role in inflammatory diseases, we hypothesize that LPS-mediated Dusp1 dephosphorylation is orchestrated by Pgam1. Our research seeks to clarify the regulatory function of Pgam1 in Dusp1 dephosphorylation and subsequent degradation, potentially disrupting MQC in cardiomyocytes and exacerbating endotoxemia-induced myocardial injury.

## Methods

### Mice

All animal experiments were conducted in compliance with the institutional guidelines of the National Institutes of Health and were authorized by the Animal Care and Use Committee of Guangdong Provincial Hospital of Chinese Medicine. Cardiomyocyte specific *Dusp1* knockout (*Dusp1^cko^*) mice, cardiomyocyte specific *Pgam1* knockout (*Pgam1^cko^*) mice, and Dusp1 transgenic (*Dusp1^Tg^*) mice were produced based on previous studies 20-23. All experimental mice were littermates or the offspring of littermates. Mice were housed 4 per cage, with a 12 h light/dark cycle, 7AM on (Z=0), 7PM off, in climate-controlled rooms. All experimental mice were either littermates or progeny of littermates. The mice were kept 4 per cage under a 12-hour light/dark cycle (lights on at 7 AM [Z=0] and off at 7 PM) in climate-controlled rooms. This treatment was administered for 48 hours, following a protocol established in our previous studies 20, 24. To inhibit the activity of Pgam1, mice were injected with PGMI-004A (10 mg/kg, Cat. No. 1313738-90-7, MedChemExpress) 12 hrs before LPS treatment.

### Echocardiography

The Vivid-q Ultrasound system (General Electric Company) fitted with a 5.0-13.0 MHz intraoperative transducer was utilized to analyze transthoracic two-dimensional echocardiography. The thickness of the anterior and posterior walls of the left ventricle was measured blindly using M-mode tracings in the parasternal short-axis perspective. The internal diameters during end-systole and end-diastole were employed to determine the ejection fraction of the left ventricle.

### Molecular docking

Docking analyses were conducted utilizing the AutoDock Vina software. The process was executed to generate a range of potential conformations and orientations for the ligand at the active site. The protein data was converted into a PDBQT file containing its structure with hydrogens added to all polar residues 25. All ligand bonds were defined as rotatable. Protein-fixed, ligand-flexible docking calculations were carried out using the Lamarckian genetic algorithm (LGA) approach. The docking location on the protein target was identified by setting up a grid box with default spacing, centered on the position of the native ligand. The optimal conformation, identified as having the lowest binding energy, was selected upon completion of the docking search. Interactions between protein-ligand complexes, including hydrogen bonds and bond lengths, were examined using PyMol.

### Mitochondrial potential assessment and mitochondrial ROS evaluation

To investigate variations in mitochondrial potential (ΔΨm), cells were labeled with JC-1 (T3168, Invitrogen) and then assessed 26. To determine mitochondrial reactive oxygen species (ROS) production, cardiomyocytes were treated thrice with PBS and incubated with 5 μM MitoSOX Red (M36008, Invitrogen), a fluorescent mitochondrial superoxide indicator, for 15 minutes. mROS were subsequently observed with an Olympus IX73 microscope, and signal intensities were measured using cellSens software 25.

### Caspase-3 activation analysis

Caspase-3 activity in heart tissues was assessed through the Apo-ONE Homogeneous Caspase-3 kit (Promega, #G7792). In brief, the caspase substrate ZDEVD-R110 was diluted 1:100 in Apo-ONE Homogeneous Caspase-3 buffer and incubated with the samples for one hour at 37 °C. Fluorescence was detected at 521 nm 27.

### qPCR

RNA was extracted from heart tissues or HL-1 cells using Trizol (Invitrogen). After adding chloroform, samples were shaken, incubated on ice, and the aqueous phase was mixed with isopropanol, followed by centrifugation. RNA pellets were washed with 75% ethanol, dried, and dissolved in nuclease-free water 28. A total of 2 μg RNA was reverse-transcribed using M-MLV Reverse Transcriptase (Invitrogen). Quantitative RT-PCR was performed using SYBR Select Master Mix (Roche) on a ViiA 7 Real-Time PCR System, with Ct values normalized to 18S rRNA. Primer details are in Supplementary Table 1.

### Cardiomyocytes isolation

Cardiomyocytes were collected from mice via a Langendorff apparatus. After 10 minutes of heparinization, hearts were excised from anesthetized mice and cannulated for retrograde perfusion with Ca^2+^-free Tyrode solution 29. The hearts were then digested and the ventricles were minced in the enzyme solution, neutralized with Ca^2+^-free Tyrode with 10% FBS, and gently pipetted to dissociate CMs. The cells were filtered through a 100 μm nylon mesh to remove debris.

### Assessment of contractility in cardiomyocytes

Cardiomyocytes were isolated from mice. Following two washes in Tyrode's buffer, cells were resuspended and maintained for 15 minutes. Contractility was assessed using the IonOptix Fluorescence Measurement and Cell Dimensioning System 30. Myocytes were subjected to 1 Hz electrical field stimulation with a pulse duration of 4 ms. Simultaneous recordings of sarcomere length and fluorescence were acquired using IonOptix IonWizard software. Fura-4 AM ratio data were background-corrected and analyzed for sarcomere shortening as previously described 31.

### Cell culture and siRNA/plasmids transfection

HL-1 cells were purchased from the ATCC. HL-1 cells were incubated with 10 μg/mL LPS for 24 hours. The cells were cultured at 37°C in a humidified 5% CO_2_ environment at a density of 4.104/cm². Medium was renewed twice a week. PGMI-004A (5 μM, Cat. No. 1313738-90-7, MedChemExpress) was placed on cells for 4 hrs minutes prior to LPS treatment. Dusp1 shRNA (Cat.No. #sc-35938-SH, Santa Cruz Biotechnology, Inc.), Pgam1 shRNA (Cat.No. #sc-152184-SH, Santa Cruz Biotechnology, Inc.), and Dusp1 plasmids (Cat.No. #sc-422507-ACT, Santa Cruz Biotechnology, Inc.) were transfected using RNAiMax (Thermo, Les Ulis, France) or Mirus LT1 reagents (Mirus-Bio, Euromedex, Souffelweyersheim, France), following the manufacturer's procedures 32. All transfections were performed in suspension before distribution in wells and subsequent poly(I:C) treatment in order to prevent transfection yield variability.

### Plasmids construct

Pgam1 and Dusp1 sequences were PCR-amplified using human cDNA as a template and cloned into either pcDNA5-His or pcDNA5-HA vectors. Additionally, based on Pgam1 and Dusp1 protein domains, truncated His-Pgam1 plasmids (ΔTM, ΔWDXNWD, ΔNXESGE, ΔPGAM) were constructed 33.

### ATP measurement

Heart tissues were homogenized with 0.5M perchloric acid (10μL/mg) using a Dounce tissue homogenizer on ice. ATP in the supernatant was quantified by colorimetric method using an ATP determination kit (Abcam, ab83355) at 570 nm wavelength and normalized to the protein level 34.

### XFe24 seahorse assay

HL-1 cells were plated in a laminin-coated XF24 plate using MEM. The medium was then replaced with sodium bicarbonate-free DMEM containing the same supplements for 1 hour. OCR was measured at 37°C using an XFe24 analyzer (Seahorse Bioscience, MA) after adding 1 μM oligomycin A, 1 μM FCCP, and 1 μM rotenone/antimycin A 35.

### Immunoblotting

Lysates were analyzed by immunoblotting on 7.5-12% SDS-PAGE, loading 50 μg of protein per lane 36. Primary antibodies were diluted in PBS-Tween with suitable blocking agents. Immunoreactive bands were detected using enhanced chemiluminescence (Amersham Pharmacia Biotech) on a LAS-4000 imaging system (FujiFilm, TDI, Madrid) after incubation with HRP-conjugated secondary antibodies 37. GAPDH served as a loading control. Details of the antibodies are provided in Supplemental Table-2.

### Statistical analyses

The figure legends and text specify the number of biological or technical replicates. For comparisons involving more than two groups, a one-way ANOVA followed by Tukey's test was applied. Data are expressed as mean ± SEM, and statistical analysis was conducted using GraphPad Prism8.

## Results

### Dusp1 is a novel biomarker of pediatric septic shock patients

**Figure 1:**
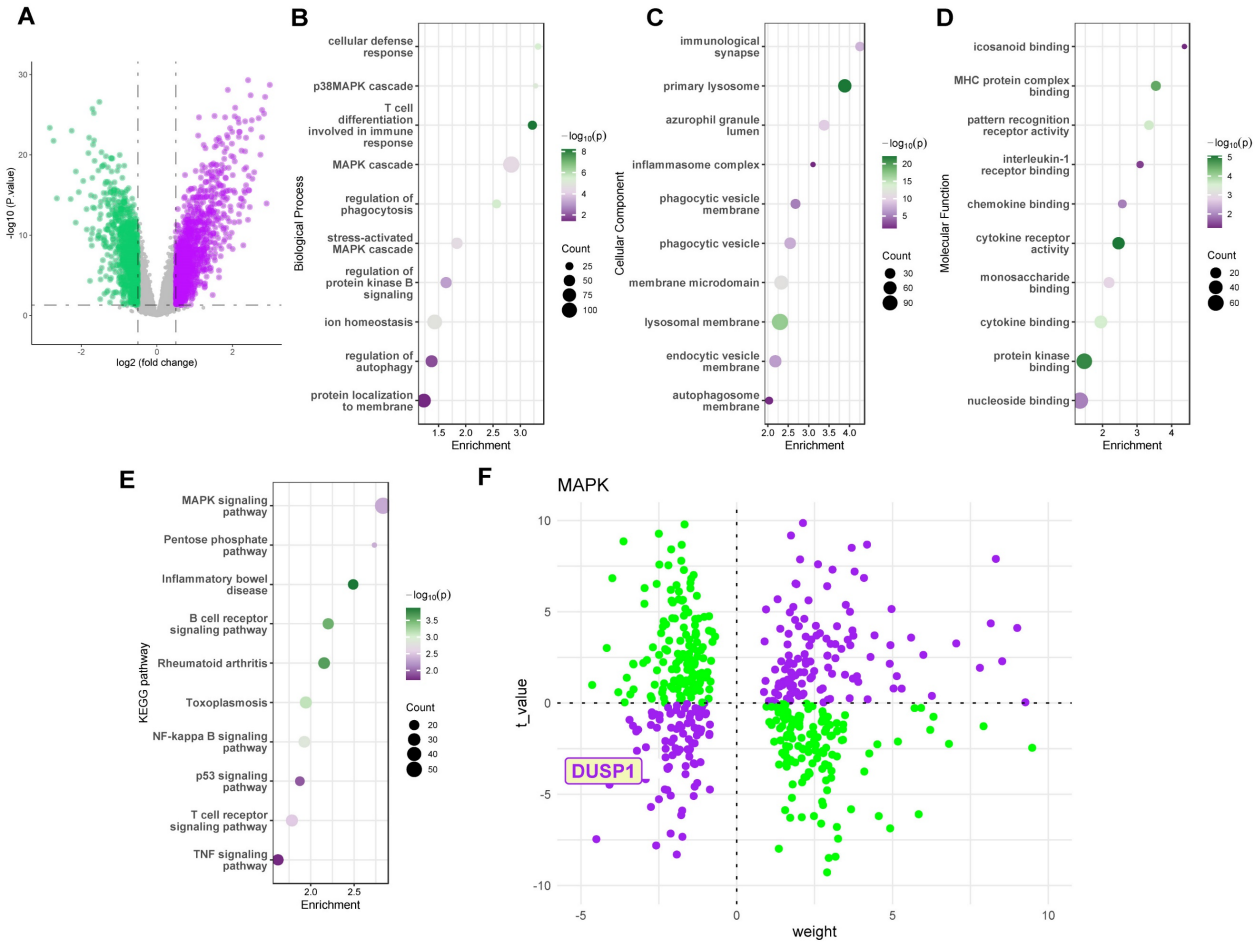
** Dusp1 is a novel biomarker of pediatric septic shock patients. A.** The volcano plot of differentially expressed genes in the GSE26440 pediatric septic shock cohort. **B-D.** GO analysis of the differentially expressed genes in the GSE26440 database. **E.** KEGG analysis of the differentially expressed genes in the GSE26440 database.** F.** The decoupleR package was used to assess the activation or inhibition weights of different differentially expressed genes.

To investigate key regulatory proteins in endotoxemia, we utilized the GSE26440 dataset, a cohort comprising peripheral blood sequencing data from 32 pediatric control patients and 98 pediatric septic shock patients. Figure 1A illustrates the volcano plot of differentially expressed genes in the GSE26440 pediatric septic shock cohort. GO and KEGG analyses of these genes (Figure 1B-E) revealed the MAPK pathway as a critical signaling pathway significantly altered in the peripheral blood of septic shock patients. Subsequently, we employed the decoupleR package to assess the activation or inhibition weights of different differentially expressed genes on the Dusp1/MAPK pathway. Results indicated that the DUSP1 gene was located in the third quadrant, distant from the origin, in the pediatric septic shock cohort (Figure 1F). This suggests that Dusp1 exhibits both a significantly negative t-value (indicating substantial downregulation of gene expression) and a high weight value (reflecting a strong inhibitory effect on the pathway) in septic shock patients.

### Dusp1 is a novel biomarker of Dusp1 is a novel biomarker of pediatric septic shock patients

**Figure 2:**
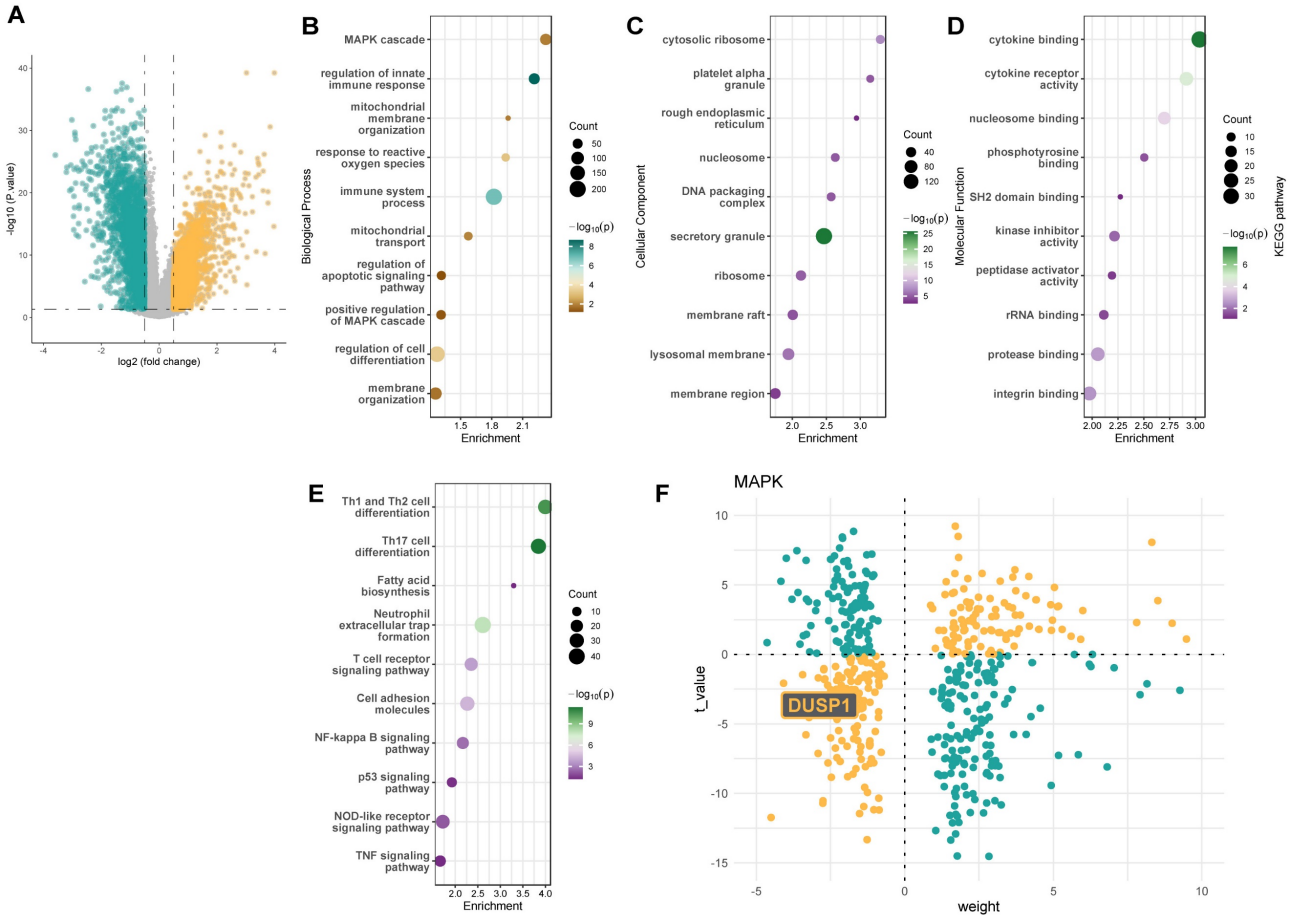
** Dusp1 is a novel biomarker of pediatric septic shock patients. A.** The volcano plot of differentially expressed genes in the GSE131761 adult septic shock cohort. **B-D.** GO analysis of the differentially expressed genes in the GSE131761database. **E.** KEGG analysis of the differentially expressed genes in the GSE131761database. **F.** The decoupleR package was used to assess the activation or inhibition weights of different differentially expressed genes.

Consistent with these findings, we further analyzed the GSE131761 adult septic shock cohort, which includes peripheral blood sequencing data from 15 control patients, 81 postoperative septic shock patients, and 33 postoperative non-septic shock patients. We selected the 15 control patients and 81 postoperative septic shock patients for differential gene analysis. Figure 2A presents the volcano plot of differentially expressed genes in the GSE131761 adult septic shock cohort. GO and KEGG analyses of these genes (Figure 2B-E) again highlighted the MAPK pathway as a key signaling pathway significantly altered in the peripheral blood of septic shock patients. The decoupleR package analysis also revealed a significantly high negative weight value for the Dusp1 gene (Figure 1F). These results collectively suggest that the Dusp1/MAPK pathway may be a critical protein in endotoxin-induced myocardial injury.

### Dusp1 is a novel biomarker of endotoxemia-mediated myocardial injury

**Figure 3:**
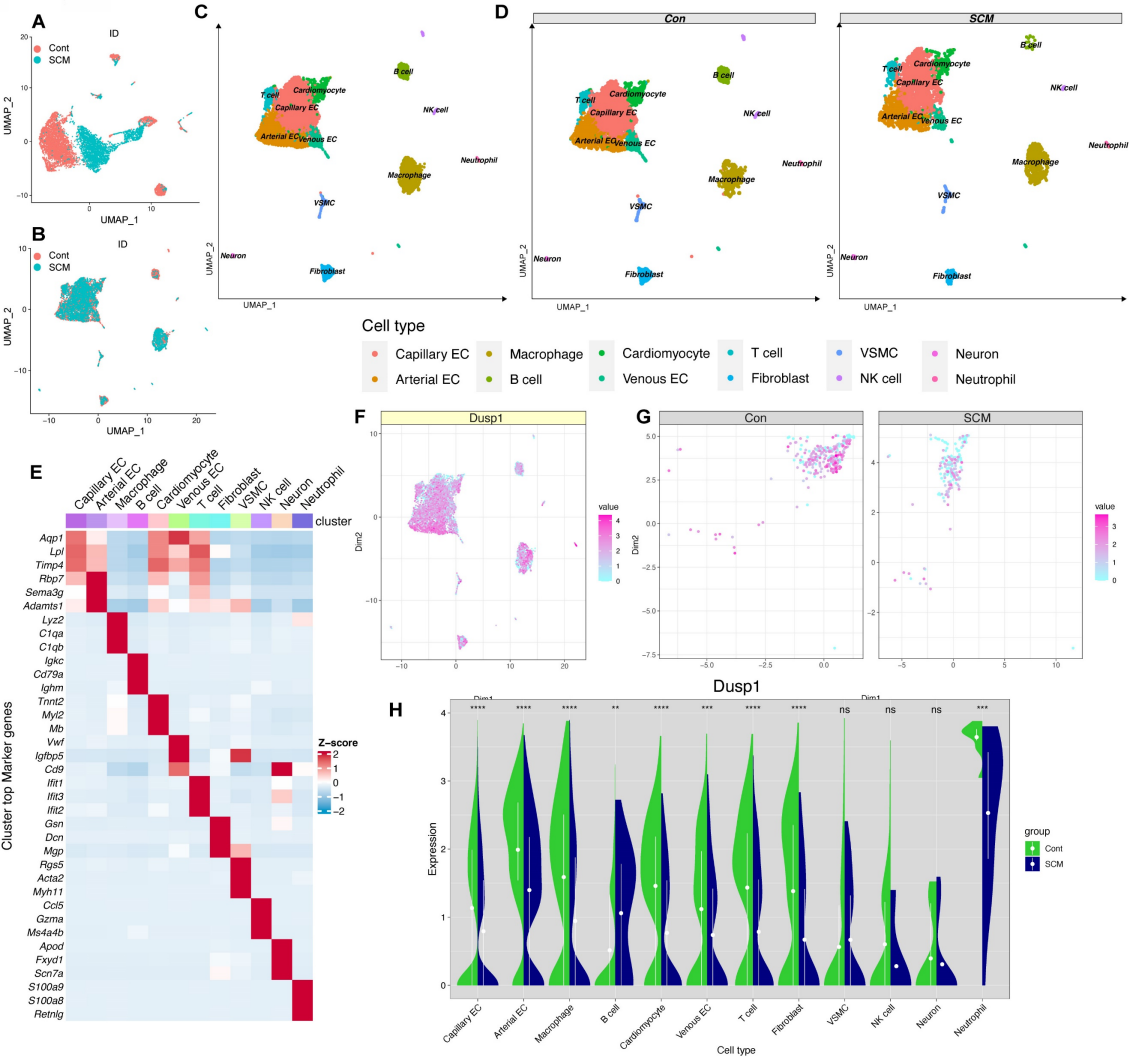
** Dusp1 is a novel biomarker of endotoxemia-mediated myocardial injury. A-B.** UMAP plots depicting the single-cell transcriptomic landscape before and after harmony integration. **C-D.** Annotated UMAP visualizations showcasing the identified cell subpopulations in the overall dataset and across experimental groups. **E.** Heatmap displaying the hypervariable genes in each cell subpopulation. **F.** Violin plots illustrating the differential expression of Dusp1 across various cell subpopulations in the control and SCM groups. **G.** Feature plots visualizing the expression levels of Dusp1 specifically within the cardiomyocyte subpopulation in the control and SCM groups. **H.** Feature plot depicting the overall distribution of Dusp1 expression across the entire scRNA-seq dataset.

Incorporating a control group mouse and an SCM model mouse cardiac single-cell dataset (CD45^+^/CD45^-^) from the single-cell dataset GSE190856, the data were merged (Figure 3A-B) and subgroups (Figure 3C-D) were annotated and displayed according to the known cell-type marker genes. Heatmap were employed to illustrate the distribution of the single gene Dusp1 in each subgroup (notably decreased in myocardial cells of SCM group) (Figure 3E). Feature plot was used to visualize the distribution of Dusp1 in each subgroup of the single gene, with a focus on extracting the myocardial subgroup (Figure 3F-G). Feature plot grouping showcased the intergroup differences of Dusp1 in the myocardial cell subgroups of the control and SCM groups (Figure 3G), with notably higher expression levels of Dusp1 represented by high-value hot spots in the control group. Violin plots were used to visualize the distribution of Dusp1 in each subgroup of the single gene, with a focus on extracting the myocardial subgroup (Figure 3H).

**Figure 4:**
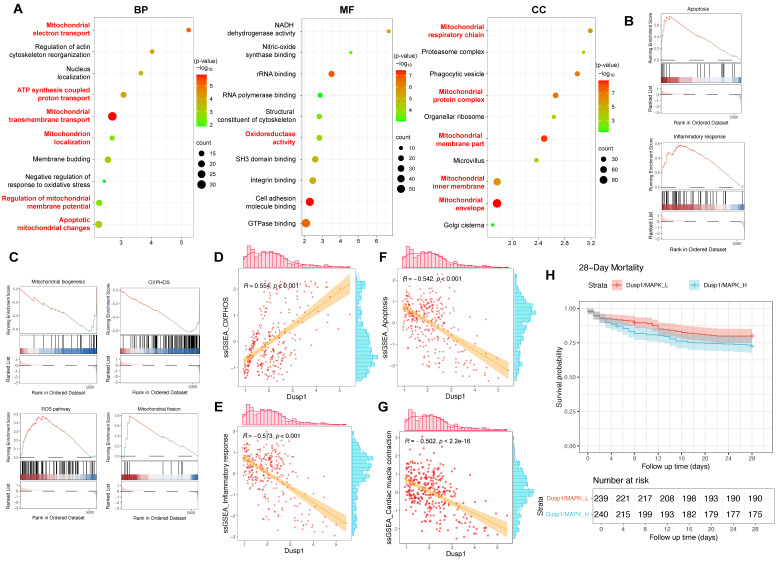
** Dusp1 is associated with the prognosis of septic patients. A.** Gene Ontology Enrichment Analysis results for the differentially expressed genes between the control and SCM groups within the cardiomyocyte subpopulation, identifying key biological processes, molecular functions, and cellular components associated with the observed transcriptomic differences. **B-C.** GSEA (Gene Set Enrichment Analysis) plots highlighting the related pathways in the control and SCM groups within the cardiomyocyte subpopulation. **D-F.** Correlation analysis investigating the relationship between Dusp1 expression levels and the activity of OXPHOS, apoptosis, and inflammatory response pathways within the cardiomyocyte subpopulation of the SCM group. **G.** ssGSEA algorithm was used to score the "Cardiac muscle contraction" gene set in cardiomyocyte subpopulations of septic cardiomyopathy mouse heart samples. **H.** The GSE65682 dataset was used to analyze the role of Dusp1/MAPK in the prognosis of septic patients.

The differential expression of genes in myocardial cells between the control and SCM subgroups was analyzed, followed by GO enrichment analysis and GSEA analysis. The GO analysis suggested a marked enrichment of disrupted genes related to mitochondria-associated cellular components (CC) and biological processes (BP) in the SCM group (Figure 4A). GSEA analysis indicated a significant upregulation of pathways such as inflammation, apoptosis, and ROS in SCM, while pathways like OXPHOS and mitochondrial biogenesis were notably downregulated (Figure 4B-C).

Each cell in the myocardial cell subgroup was scored using ssGSEA to evaluate the activation status of relevant pathways in individual cells and correlate these with the corresponding expression levels of Dusp1. The analysis aimed to determine whether Dusp1 significantly elicited agonistic or antagonistic effects on the aforementioned pathways. The expression of Dusp1 was positively correlated with the activity of the OXPHOS pathway in the myocardial cell subgroup, while it exhibited a significant negative correlation with the activity of inflammatory and apoptotic pathways, suggesting a potential antagonistic mechanism at play (Figure 4D-G). Furthermore, we employed the ssGSEA algorithm to score the "Cardiac muscle contraction" gene set in cardiomyocyte subpopulations of septic cardiomyopathy mouse heart samples. Correlation analysis between Dusp1 expression and "Cardiac muscle contraction" gene set scores in the cardiomyocyte subpopulation revealed a significant negative correlation (Figure 4D-G). These results confirm Dusp1 as a potential key factor in endotoxemia-induced myocardial injury.

To further validate the association of Dusp1 with myocardial injury, we utilized the GSE65682 dataset, a sepsis cohort sequencing dataset with 28-day survival data. This dataset encompasses 802 blood samples, including 760 from septic patients. After excluding samples without clinical outcome data, 479 sepsis data points were obtained. Using ssGSEA, each patient was scored for Dusp1/MAPK pathway activity, and patients were divided into "Dusp1/MAPK_L" (low activity) and "Dusp1/MAPK_H" (high activity) groups based on the median score. A survival model was then constructed. Results showed significant differences in prognosis between the two groups, with survival time significantly decreased in the Dusp1/MAPK_L group (Figure 4H), suggesting a significant positive correlation between Dusp1/MAPK pathway activity and poor prognosis (28-day mortality) in septic patients.

### *Dusp1* deficiency is associated with blunted heart function

**Figure 5:**
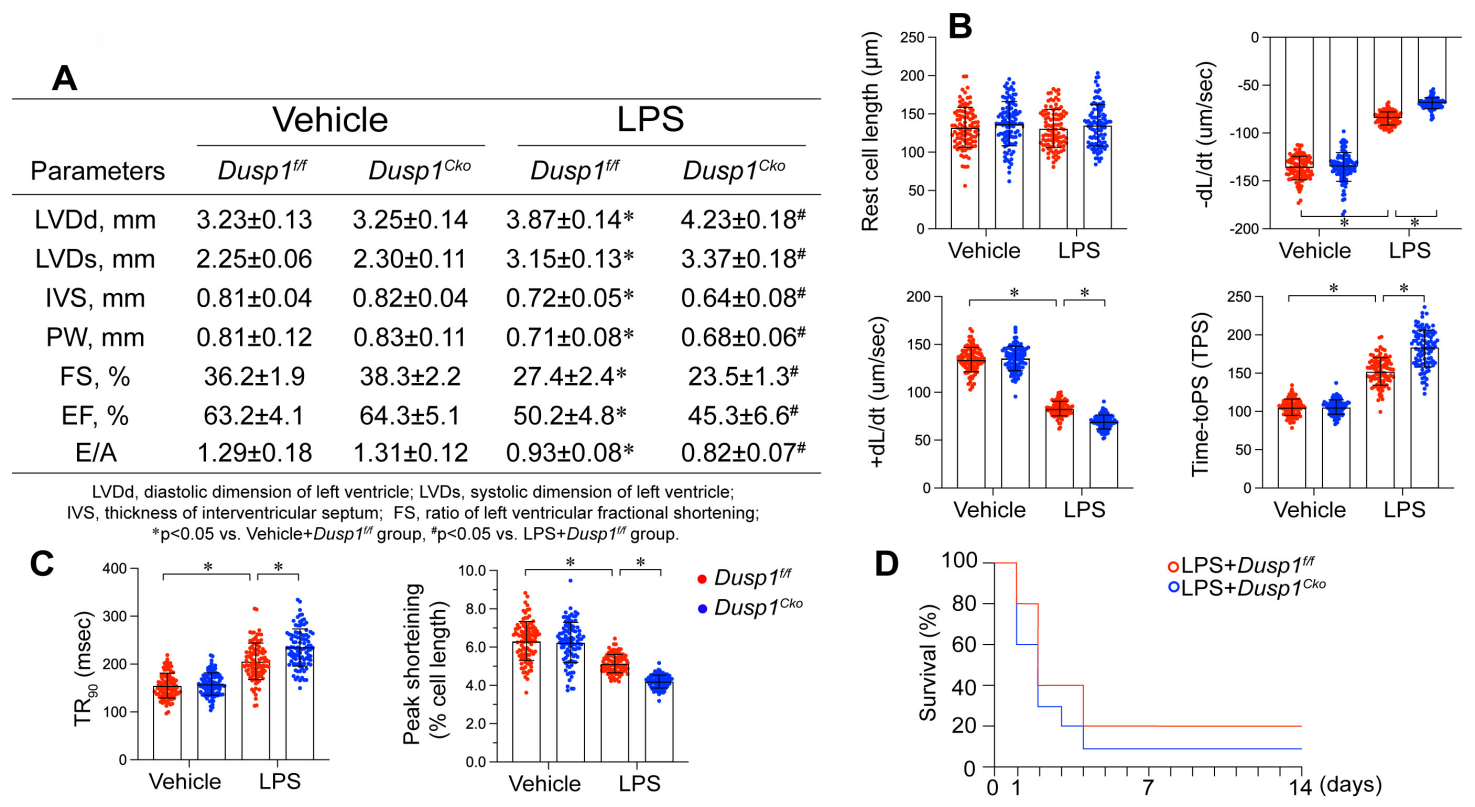
***Dusp1* deficiency is associated with blunted heart function.** Cardiomyocyte-specific *Dusp1* knockout (*Dusp1^cko^*) mice and its control literature *Dusp1^f/f^* mice were injected with lipopolysaccharide (LPS) at 10 mg/kg for 48 hrs to induce an endotoxemia myocardial model. Single cardiomyocytes were isolated from *Dusp1^cko^* mice and *Dusp1^f/f^* mice on a Langendorff apparatus and the mechanical properties of cardiomyocytes were measured. **A.** Echocardiography was used to determine cardiac function. **B-C** Mechanical properties were measured in 30-40 cardiomyocytes per group. **D.** Survival data for *Dusp1^cko^* mice and *Dusp1^f/f^* mice. *p<0.05.

To elucidate the pathophysiological consequences of Dusp1 downregulation in endotoxemia-induced myocardial dysfunction, we engineered cardiomyocyte-specific *Dusp1* knockout (*Dusp1^cko^*) mice and established a lipopolysaccharide (LPS)-induced endotoxemia myocardial model. Echocardiographic assessment revealed that LPS exposure impaired systolic function in *Dusp1^f/f^* mice, as featured by reduced left ventricular ejection fraction (LVEF), fractional shortening ratio (FS), and increased systolic dimension (LVDs) (Figure 5A). These functional deficits were further exacerbated in LPS-treated *Dusp1^cko^* mice (Figure 5A). Diastolic function, assessed by the ratio of early to late transmitral flow velocities (E/A) and left ventricular diastolic dimension (LVDd), was also compromised in *Dusp1^f/f^* mice and showed further deterioration in *Dusp1^cko^* mice under endotoxemic conditions (Figure 5A). To demonstrate the cardioprotective role of Dusp1 against endotoxemia, we also generated the *Dusp1* transgenic (*Dusp1^Tg^*) mice. Compared to wild-type (WT) mice, *Dusp1^Tg^* mice exhibited resistance to LPS-mediated suppression of myocardial systolic and diastolic functions (Supplemental Figure 1A).

To further observe the cardiomyocyte cardiomyocyte contractility and relaxation *ex vivo*, we also collected single cardiomyocytes from mice following LPS treatment. LPS exposure suppressed contractile parameters, including peak shortening (PS), maximal velocity of shortening (+dL/dt), and time-to-peak shortening (TPS) in *Dusp1^f/f^*-derived cardiomyocytes, with further deterioration observed in *Dusp1^cko^* cardiomyocytes (Figure 5B-C). In contrast, cardiomyocytes from *Dusp1^Tg^* mice demonstrated resilience to LPS-induced contractile impairments when compared with those from WT mice (Supplemental Figure 1B). Moreover, relaxation parameters, such as maximal velocity of relengthening (-dL/dt) and time-to-90% relengthening (TR90), were adversely affected in LPS-treated *Dusp1^f/f^* cardiomyocytes, an effect exacerbated in *Dusp1^cko^* mice (Figure 5B-C) but mitigated in *Dusp1^Tg^* mice (Supplemental Figure 1B). The impaired cardiac function in LPS-treated *Dusp1^cko^* mice was associated with a further reduction in survival rate compared to LPS-treated *Dusp1^f/f^* mice (Figure 5D). Conversely, the LPS-shortened survival rate was prolonged in *Dusp1^Tg^* mice compared to WT mice (Supplemental Figure 1C).

### Dusp1 overexpression attenuates inflammatory response and cardiomyocyte death

**Figure 6:**
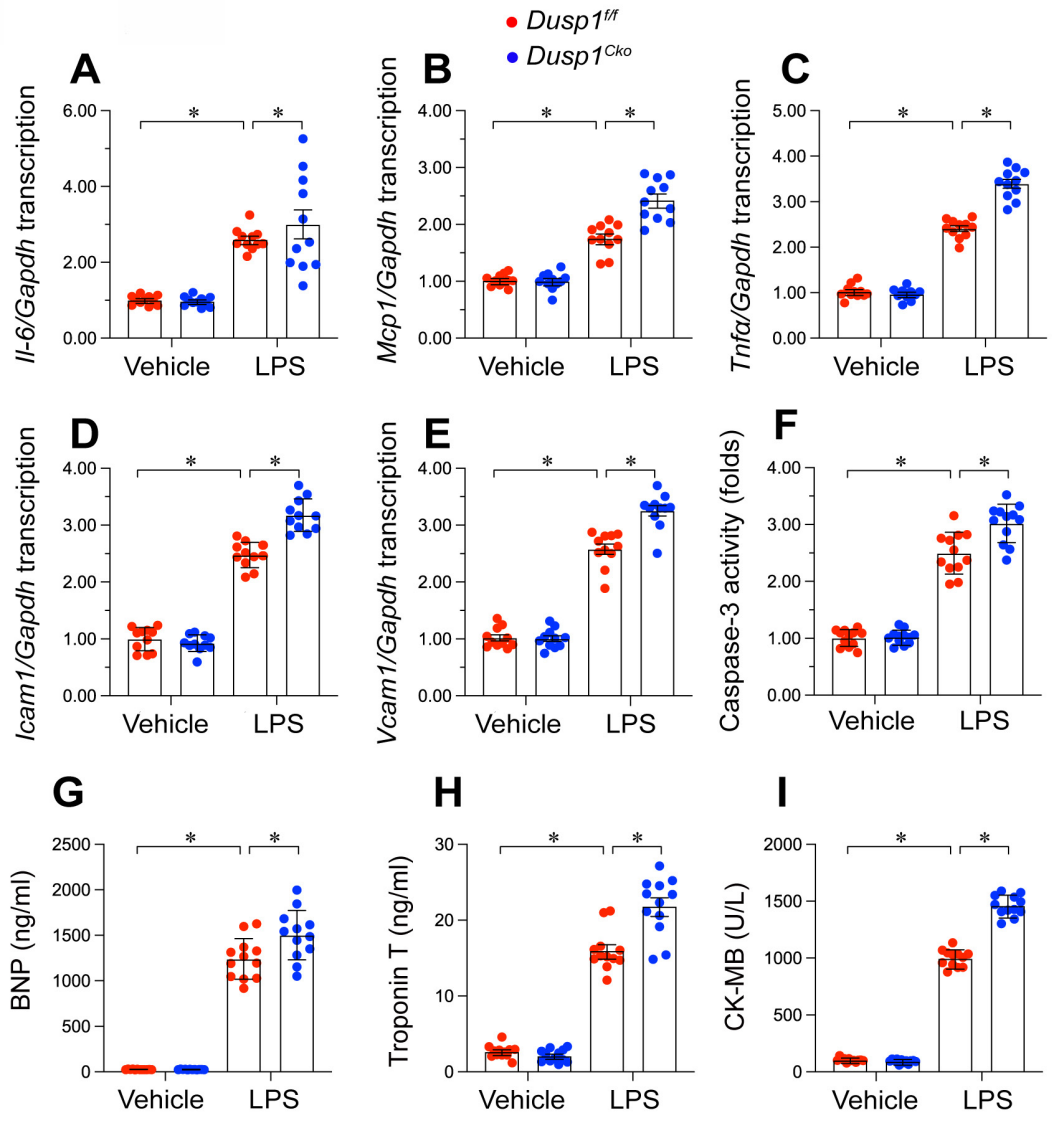
***Dusp1* deficiency augments LPS-induced inflammation response and cardiomyocyte death.** Cardiomyocyte-specific *Dusp1* knockout (*Dusp1^cko^*) mice and its control literature *Dusp1^f/f^* mice were injected with lipopolysaccharide (LPS) at 10 mg/kg for 48 hrs to induce an endotoxemia myocardial model.** A-C.** RNA were isolated from heart tissues and the transcription of* Il-6*, *Mcp1*, and *Tnfα* were detected by qPCR. **D-E.** RNA were isolated from heart tissues and the transcription of *Icam1* and *Vcam1* were measured by qPCR. **F.** ELISA kit was used to detect the activity of caspase-3 in heart tissues.** G-I.** Serum were collected from mice after LPS exposure and the concentrations of BNP, TnT, and CK-MB were analyzed by ELISA. Data are shown as mean ± SEM. In each group, four animals or four independent cell isolations were used. Each experiment was conducted with three replicates and the dots in each panel represent the outcomes of these replicate experiments. *p<0.05.

Inflammation and cardiomyocyte death have been identified as key molecular mechanisms underlying endotoxemia-induced myocardial damage. Based on this, we specifically examined how *Dusp1* deficiency or overexpression affects cardiac injury in an endotoxemic context. Quantitative PCR (qPCR) analysis of pro-inflammatory cytokines demonstrated that LPS significantly increased mRNA levels of *Il-6*, *Mcp1*, and *Tnfα* in both *Dusp1^f/f^* (Figure 6A-C) and WT mice (Supplemental Figure 2A-C) compared to baseline. However, these inflammatory transcripts were further upregulated in *Dusp1^cko^* mice (Figure 6A-C), whereas they returned to near-normal levels in *Dusp1^Tg^* mice under LPS exposure (Supplemental Figure 2A-C).

Moreover, the transcription of adhesion molecules, namely *Icam1* and *Vcam1*, was rapidly elevated by LPS in the hearts of *Dusp1^f/f^* mice (Figure 6D-E), with further increases observed in *Dusp1^cko^* mice (Figure 6D-E). Conversely, *Dusp1* overexpression in *Dusp1^Tg^* mice significantly countered the LPS-induced upregulation of these molecules compared to WT mice (Supplemental Figure 2D-E).

Cardiomyocyte apoptosis was measrued by caspase-3 activity experiment. Caspase-3 activity, as measured by ELISA, was elevated in the hearts of *Dusp1^f/f^* (Figure 6F) and WT mice (Supplemental Figure 2F) following LPS treatment, with further augmentation in *Dusp1^cko^* mice (Figure 6F) and attenuation in *Dusp1^Tg^* mice (Supplemental Figure 2F).

To evaluate *in vivo* cardiomyocyte damage, serum levels of biomarkers, including brain natriuretic peptide (BNP), troponin T (TnT), and creatine kinase-MB (CK-MB), were measured. LPS administration significantly increased these markers in *Dusp1^f/f^* mice, with more pronounced effects observed in *Dusp1^cko^* mice (Figure 6G-I). In contrast,* Dusp1^Tg^* mice showed no significant LPS-induced elevation in these biomarkers compared to WT mice (Supplemental Figure 2G-I). Collectively, these findings substantiate the necessity of Dusp1 abundance in maintaining cardiomyocyte functionality and mitigating inflammatory responses in endotoxemia-induced cardiac dysfunction.

### LPS induced Dusp1 downregulation through suppressing Dusp1 phosphorylation

**Figure 7:**
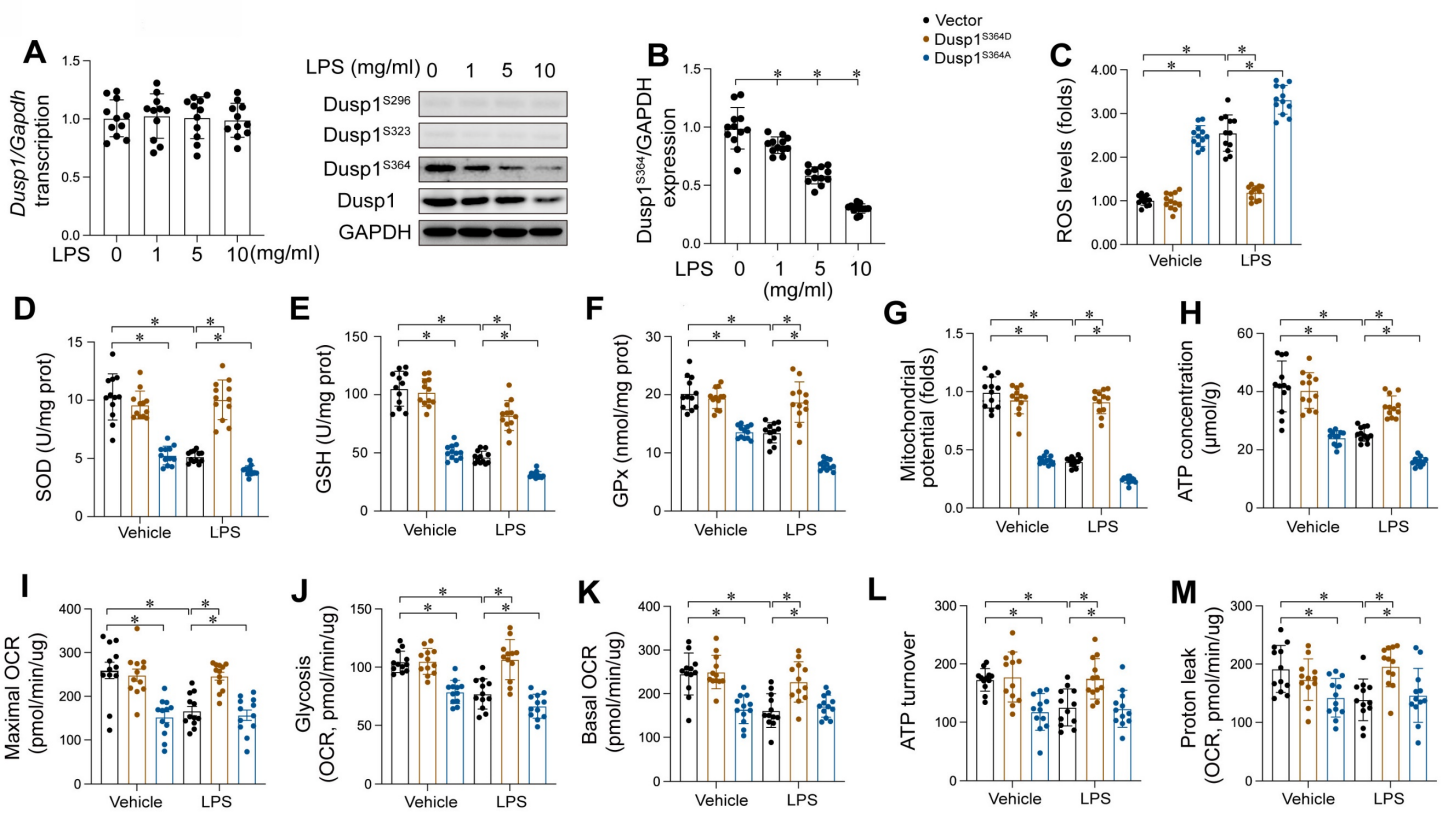
** LPS induced Dusp1 downregulation through suppressing Dusp1 phosphorylation. *WT* mice were injected with lipopolysaccharide (LPS) at 10 mg/kg for 48 hrs to induce an endotoxemia myocardial model. HL-1 cells were treated with LPS at 10 μg/mL for 24 hrs. A.** qPCR analysis of Dusp1 transcription in heart tissues at different doses of LPS. **B.** Proteins were isolated from heart tissues and the western blots were used to analyze the expression of p-Dusp1^Ser296^, p-Dusp1^Ser323^, and p-Dusp1^Ser364^. **C.** Mitochondrial ROS were measured via immunofluorescence. **D-F.** ELISA kits were used to measure the concentration of GSH, GPX and SOD in HL-1 cells upon LPS exposure. **G.** Mitochondrial membrane potential was determined by JC-1 probe. **H.** ATP production was measured by ELISA in HL-1 cells upon LPS exposure. **I-M.** Seahorse XF Analyzer was used to measure the mitochondrial oxygen consumption rates (OCR). Experiments were repeated at least three times. Data are shown as mean ± SEM. In each group, four animals or four independent cell isolations were used. Each experiment was conducted with three replicates and the dots in each panel represent the outcomes of these replicate experiments. *p<0.05.

Our findings substantiate the cardioprotective role of Dusp1 against myocardial injury induced by endotoxemia. However, the molecular intricacies of how LPS mediates Dusp1 downregulation in cardiomyocytes remain elusive. qPCR analysis indicated that Dusp1 transcription remains unaltered in response to LPS (Figure 7A), indicating the involvement of post-transcriptional regulation mechanisms. Previous studies have highlighted the critical influence of post-transcriptional phosphorylation on Dusp1 stability 13, with reduced phosphorylation resulting into its downregulation. Based on these findings, we hypothesized that LPS might affect Dusp1 stability via post-transcriptional dephosphorylation. To identify the phosphorylation sites critical for Dusp1 stability, we performed western blot analysis, which revealed that phosphorylation at Ser296 and Ser323 remained largely unaffected by LPS (Figure 7B), while phosphorylation at Ser364 was significantly decreased.

Mitochondria function as an important contributor in cardiomyocyte performance, primarily through ATP synthesis, rendering mitochondrial impairment a critical early indicator of endotoxemia-induced cardiomyocyte dysfunction. In our study, HL-1 cardiomyocytes were transfected with either a Dusp1 phosphorylation-disabled mutant (Dusp1^S364A^) or a phosphorylation-mimicking mutant (Dusp1^S364D^) before exposure to LPS. Mitochondrial reactive oxygen species (mROS) levels were significantly elevated in LPS-treated control HL-1 cells, an effect notably mitigated by Dusp1^S364D^ transfection (Figure 7C). Concomitantly, a marked reduction in anti-oxidative enzymes, including GSH, SOD and GPX, was observed in control cardiomyocytes, which was effectively restored in Dusp1^S364D^-transfected cells under LPS treatment (Figure 7D-F). Furthermore, LPS exposure disrupted mitochondrial potential in control cardiomyocytes, a deleterious effect attenuated by Dusp1^S364D^ transfection (Figure 7G). Given the critical role of mitochondrial membrane potential in regulating cellular ATP production, its reduction in control cells correlated with a decrease in ATP levels following LPS treatment (Figure 7H). However, transfection with Dusp1^S364D^ maintained ATP production under these conditions (Figure 7H). Utilizing a Seahorse XF Analyzer, we evaluated the mitochondrial oxygen consumption rates (OCR) in cardiomyocytes in response to different Dusp1 phosphorylation states post-LPS exposure (Figure 7I-M). Mitochondrial respiration, impaired in control cells by LPS, was normalized by Dusp1^S364D^ transfection (Figure 7I-M). In summary, our data elucidate the pivotal role of phosphorylated Dusp1 in preserving mitochondrial redox homeostasis and respiratory function during endotoxemic stress.

### Dusp1 phosphorylation improves MQC

**Figure 8:**
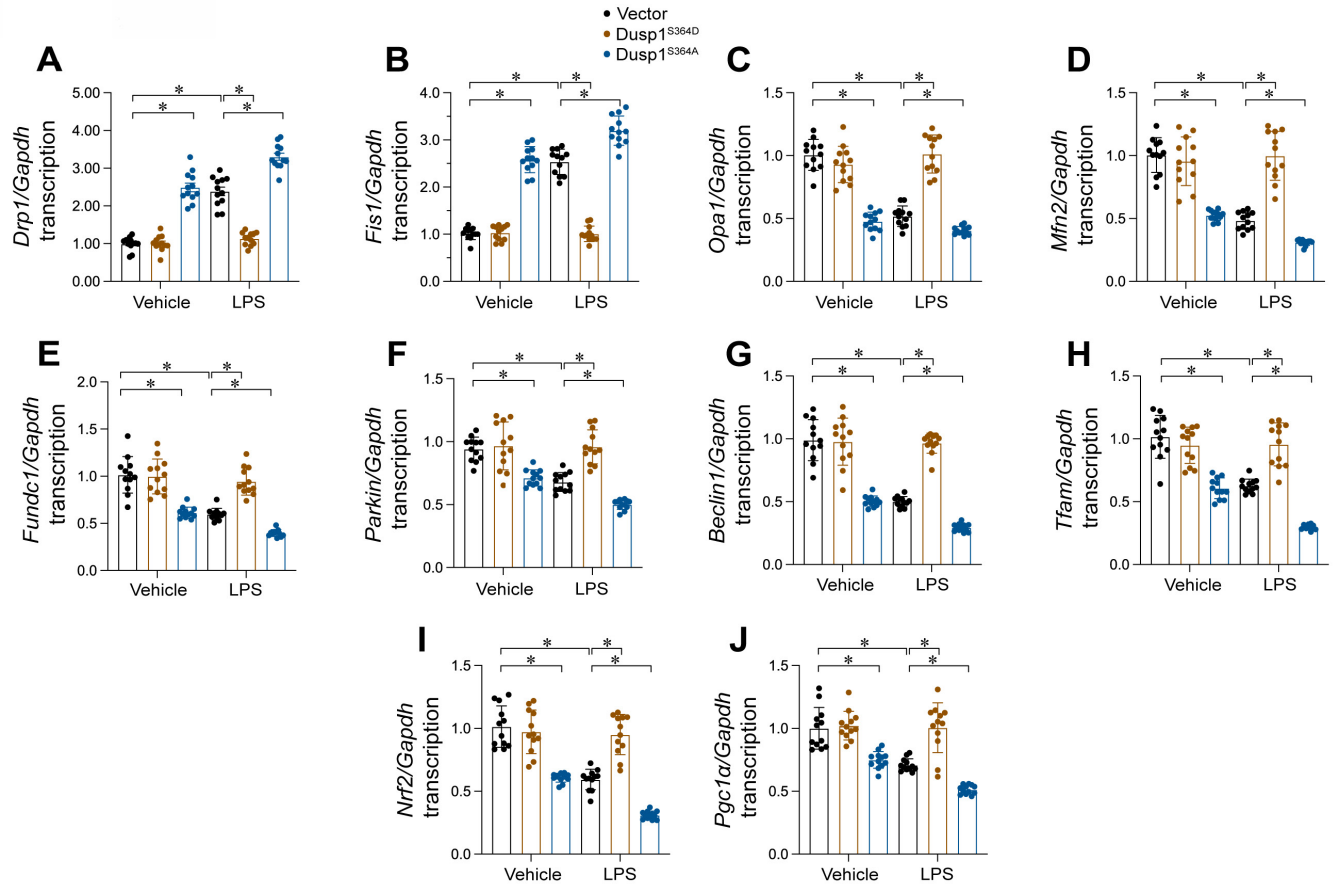
** Dusp1 phosphorylation improves MQC. HL-1 cells were transfected with Dusp1 phosphorylation-disabled mutant (Dusp1^S364A^) or a phosphorylation-mimicking mutant (Dusp1^S364D^) before exposure to 10 μg/mL LPS for 24 hrs. A-D.** RNA was isolated from HL-1 cells and then the transcription of *Drp1*, *Fis1*, *Mfn2* and *Opa1*. **E-G.** RNA was isolated from HL-1 cells and then the transcription of *Parkin*, *Fundc1*, and *Beclin1.* **H-J.** qPCR analysis of the transcription of genes related to mitochondrial biogenesis including *Pgc1α*, *Tfam*, and *Nrf2.* Experiments were repeated at least three times. Data are shown as mean ± SEM. In each group, four animals or four independent cell isolations were used. Each experiment was conducted with three replicates and the dots in each panel represent the outcomes of these replicate experiments. *p<0.05.

MQC mechanisms, encompassing mitochondrial dynamics, mitophagy, and biogenesis, are vital for preserving mitochondrial functionality. In this study, we investigated whether these processes are modulated by the phosphorylation status of Dusp1. HL-1 cells were transfected with either a phosphorylation-mimicking Dusp1 mutant (Dusp1^S364D^) or a non-phosphorylatable mutant (Dusp1^S364A^) and subjected to LPS exposure. qPCR analysis revealed that LPS caused a rise in mitochondrial fission markers *Drp1* and *Fis1*, and a reduction in fusion proteins *Mfn2* and *Opa1* (Figure 8A-D), indicating enhanced fission and reduced fusion in response to LPS. However, this imbalance was undetectable in HL-1 cells expressing Dusp1^S364D^ (Figure 8A-D).

Regarding mitophagy, qPCR showed that LPS exposure repressed levels of mitophagy-related genes (*Parkin*, *Fundc1*, and *Beclin1*), which were restored in cells expressing Dusp1^S364D^ (Figure 8E-G). Lastly, mitochondrial biogenesis, primarily regulated at the transcriptional level, was assessed through mRNA expression analysis. We observed a significant downregulation of biogenesis-related genes *Pgc1α*, *Tfam*, and *Nrf2* in LPS-treated HL-1 cells (Figure 8H-J), while the levels of these genes was restored in cells transfected with Dusp1^S364D^ transfection. Altogether, our findings illustrate that Dusp1 phosphorylation is pivotal in maintaining MQC processes, including dynamics, mitophagy, and biogenesis, under endotoxemic conditions.

### Pgam1 interacts with Dusp1

**Figure 9:**
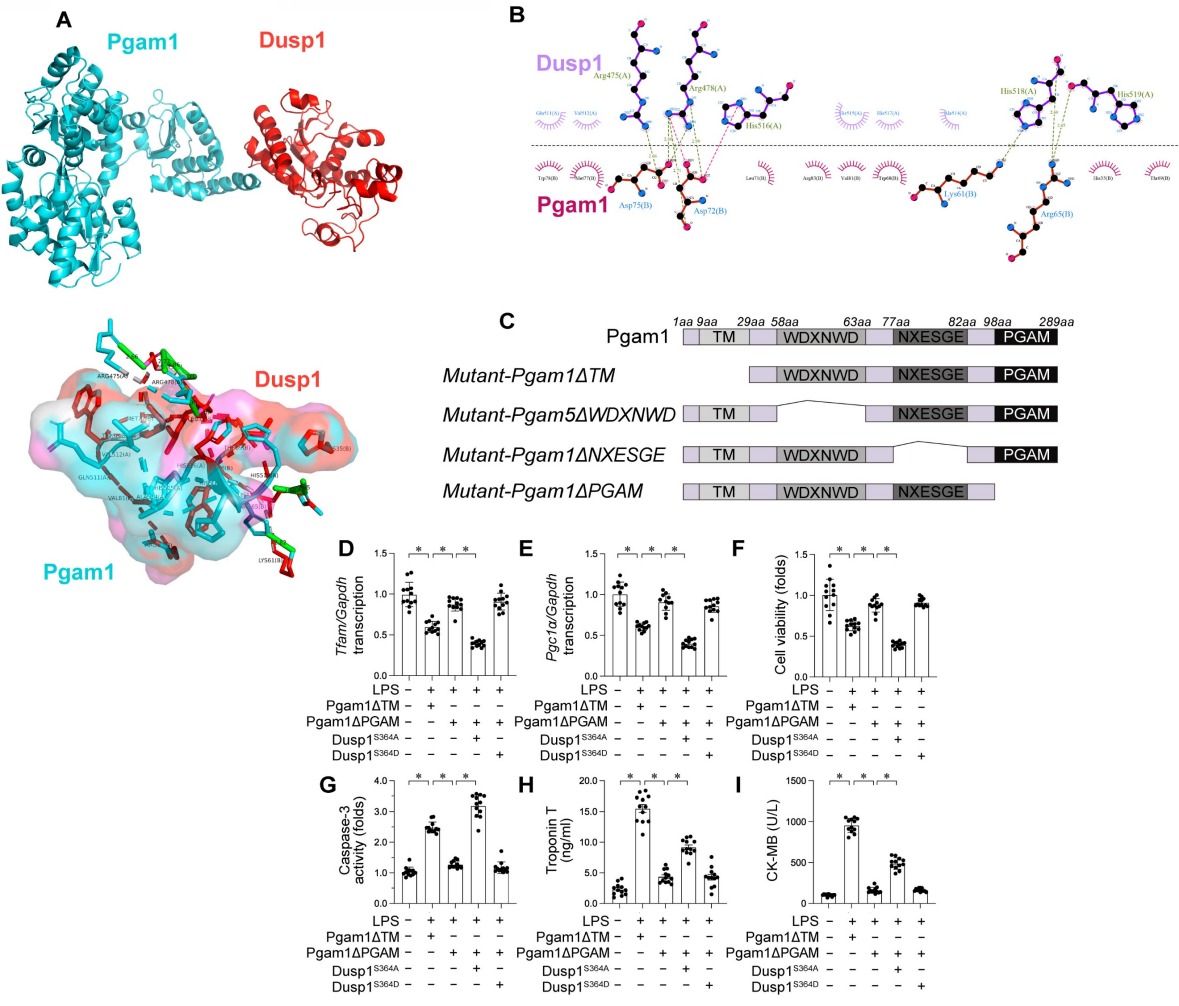
** Dusp1 is interacted by Pgam1. A-B.** Molecular docking of Dusp1 and Pgam1. **C.** Mapping of regions in Pgam1. **D-E.** HL-1 cells were transfected with HA-Pgam1ΔPGAM, HA-Pgam1ΔTM, Dusp1^S364A^, or Dusp1^S364D^ before exposure to 10 μg/mL LPS for 24 hrs. qPCR analysis of the transcription of genes related to mitochondrial biogenesis including *Pgc1α* and *Tfam***. F.** Cell viability was determined by MTT assay. **G.** ELISA analysis of caspase-3 activity in HL-1 cells. **H-I.** The concentration of TnT and CK-MB were measured in the medium of HL-1 cells. *p<0.05.

To identify the regulator of Dusp1 dephosphorylation and elucidate the mechanism underlying Dusp1 degradation in the presence of LPS, we performned a molecular docking assay which identified the active regions necessary for Dusp1-Pgam1 interaction, with a calculated binding energy of -23.1 kcal·mol^-1^ (Figure 9A-B), highlighting the specificity of this interaction.

To delineate the molecular basis of the Pgam1-Dusp1 interaction, we investigated the domains necessary for cross-linking (Figure 9C). To elucidate the influence of Pgam1/Dusp1 interaction on mitochondrial function and cardiomyocyte viability during endotoxemic stress, HL-1 cardiomyocytes were transfected with HA-Pgam1ΔPGAM and various Dusp1 mutants prior to LPS exposure. LPS-mediated transcriptional repression of mitochondrial biogenesis genes *Pgc1α* and *Tfam* was normalized by HA-Pgam1ΔPGAM or Dusp1^S364D^ transfection, but this effect was abolished in cells expressing Dusp1^S364A^ (Figure 9D-E).

Regarding cardiomyocyte viability, MTT assays indicated a decline in cell viability and an increase in caspase-3 activity following LPS treatment. Transfection with HA-Pgam1ΔPGAM preserved cardiomyocyte viability (Figure 9F) and inhibited caspase-3 activation (Figure 9G), effects that were suppressed by concurrent transfection with Dusp1^S364A^. ELISA analysis of cardiac injury biomarkers corroborated these findings, showing elevated levels post-LPS treatment, which were ameliorated by HA-Pgam1ΔPGAM transfection, but not in the presence of Dusp1^S364A^ (Figure 9H-I). In conclusion, our results underscore the protective role of Pgam1/Dusp1 disassociation in preserving MQC and cardiomyocyte function under endotoxemic conditions, a process intricately linked to the preservation of Dusp1 phosphorylation.

### Pgam1 inhibition attenuated endotoxemia-mediated myocardial damage

**Figure 10:**
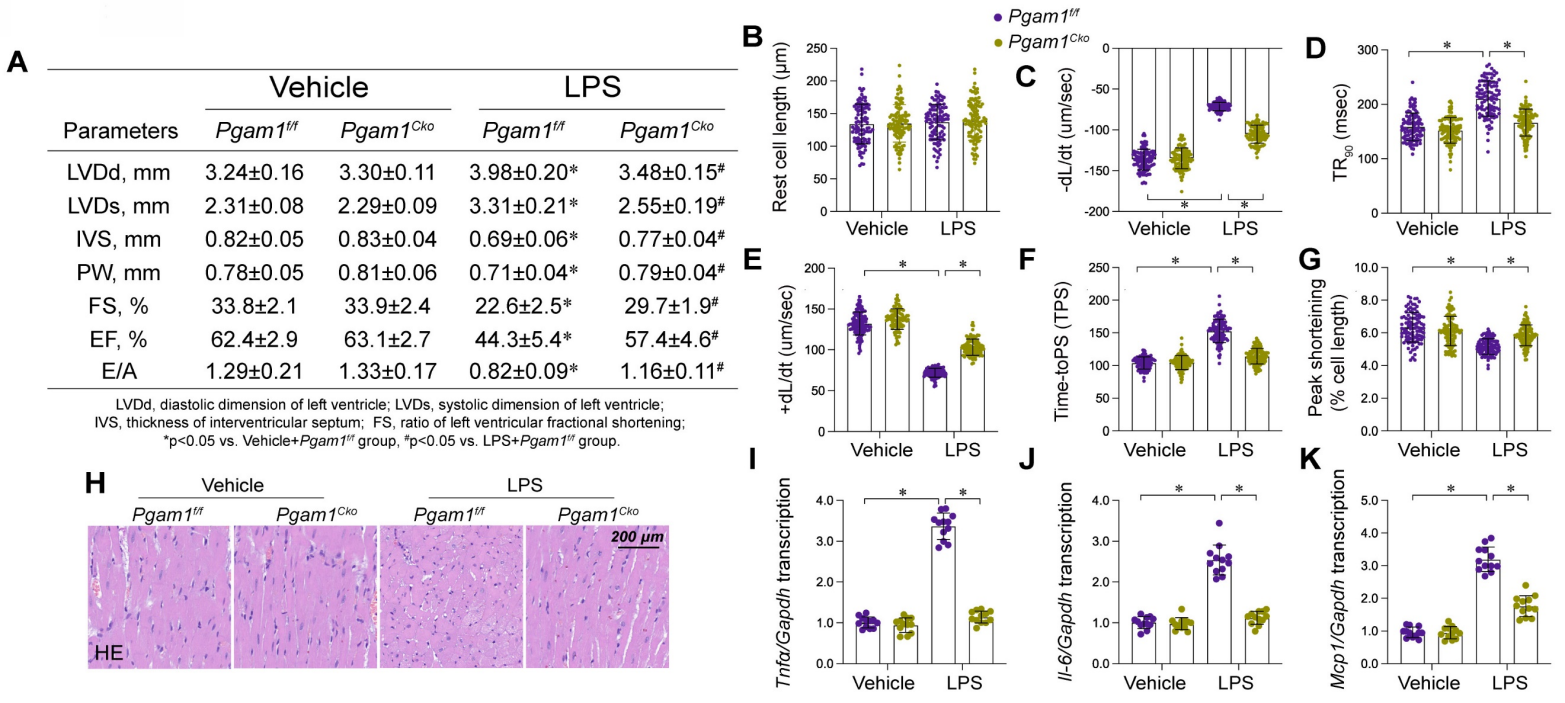
** Pgam1 deletion attenuated endotoxemia-mediated myocardial damage. Cardiomyocyte-specific *Pgam1* knockout (*Pgag5^cko^*) mice and its control literature *Pgam1^f/f^* mice were injected with lipopolysaccharide (LPS) at 10 mg/kg for 48 hrs to induce an endotoxemia myocardial model. Single cardiomyocytes were isolated from *Pgam1^cko^* mice and *Pgam1^f/f^* mice on a Langendorff apparatus and the mechanical properties of cardiomyocytes were measured. A.** Echocardiography was used to determine cardiac function. **B-G.** Mechanical properties were measured in 100-120 cardiomyocytes per group. **H.** Presentative pictures of HE staining in heart tissues after LPS exposure. **I-K.** RNA were isolated from heart tissues and the transcription of *Il-6, Mcp1*, and *Tnf*α were detected by qPCR. Data are shown as mean ± SEM. In each group, four animals or four independent cell isolations were used. Each experiment was conducted with three replicates and the dots in each panel represent the outcomes of these replicate experiments. *p<0.05.

To translate our cellular findings into an animal experiment, we employed cardiomyocyte-specific *Pgam1* knockout (*Pgam1^cko^*) mice subjected to LPS-induced endotoxemia. Echocardiographic evaluation revealed significant impairments in both systolic and diastolic cardiac functions in *Pgam1^f/f^* mice post-LPS exposure (Figure 10A). Remarkably, these functional deficits were substantially alleviated in *Pgam1^cko^* mice subjected to LPS (Figure 9A). Concordantly, LPS impaired the mechanical properties in cardiomyocytes isolated from *Pgam1^f/f^* mice, but not in those from *Pgam1^cko^* mice (Figure 10B-G). Histological examinations using HE staining unveiled that LPS-induced myofibrillar disarray and myocardial edema were mitigated following *Pgam1* ablation (Figure 10H). Furthermore, qPCR analysis unveiled a notable upregulation of inflammatory cytokines in *Pgam1^f/f^* mice in response to LPS, an effect markedly abrogated in *Pgam1^cko^* mice (Figure 10I-K). In line with this, the expression of adhesion molecules, including ICAM1 and VCAM1, escalated rapidly in the hearts of *Pgam1^f/f^* mice following LPS treatment, whereas such upregulation was absent in *Pgam1^cko^* mice (Figure 9L-N).

In addition to genetic ablation of *Pgam1*, pharmacological inhibition via PGMI-004A administration also conferred cardioprotection (Supplemental Figure 3A) and normalized cardiomyocyte performance (Supplemental Figure 3B-G) in the context of LPS-induced endotoxemic stress. Moreover, PGMI-004A attenuated the inflammatory response (Supplemental Figure 3H-J) and caused a reduction in cardiac injury biomarkers (Supplemental Figure 3K-M). Collectively, these findings substantiate the hypothesis that *Pgam1* deletion or inhibition confers cardioprotection against endotoxemic insult.

## Discussion

Our research underscores the imperative to deepen our understanding of the molecular intricacies underlying myocardial injury in endotoxemia. Current knowledge gaps significantly impede early detection and the development of targeted therapeutic strategies for this cardiac condition. Employing an array of genetically modified murine models, including *Dusp1* knockout (*Dusp1^cko^*), *Dusp1* transgenic (*Dusp1^Tg^*), and *Pgam1* knockout (*Pgam1^cko^*), our study sought to elucidate the consequences of dysregulated Pgam1, post-transcriptional dephosphorylation of Dusp1, and impaired MQC on myocardial function in the context of LPS-induced endotoxemia. Our investigation culminated in four pivotal findings. First, Pgam1 and Dusp1 have emerged as potential biomarkers for myocardial dysfunction in endotoxemia, offering fresh perspectives on the biomolecular basis of this affliction. Second, LPS exposure instigates Dusp1 degradation via post-transcriptional dephosphorylation. This dephosphorylated state of Dusp1 significantly exacerbates cardiomyocyte dysfunction and disrupts MQC, marking a novel molecular pathway implicated in cardiac impairment during endotoxemia. Third, LPS stimulation induces Pgam1 upregulation, which subsequently interacts with Dusp1. This interaction provides a new mechanistic insight into the molecular mechanism of myocardial injury under endotoxemic conditions. Fourth, the ablation of Pgam1 offers cardiac protection in the face of endotoxemic stress. However, this protective effect is attenuated by Dusp1 dephosphorylation, indicating a complex interplay affecting cardiac response to endotoxemia. In conclusion, our findings suggest that elevated Pgam1 expression in conjunction with dephosphorylated Dusp1 constitutes potential molecular underpinnings of myocardial dysfunction in endotoxemia, mainly through the perturbation of MQC. Consequently, therapeutically targeting the Pgam1/Dusp1 axis emerges as a promising strategy to ameliorate cardiac function in endotoxemia-afflicted patients.

Originally identified as a regulator of the MAPK pathway and termed mitogen-activated protein kinase phosphatase-1 (MKP-1), Dusp1 has subsequently been recognized for its broader role in modulating inflammatory responses across various disease contexts. A notable example is observed in C. difficile-induced colonic inflammation, where abnormal Dusp1 levels activates the NF-κB 38, leading to an upsurge of pro-inflammatory cytokines IL-1β and TNFα. This effect has been corroborated by studies in mouse models of septic peritonitis, which show that *Dusp1* knockout (*Dusp1^-/-^*) mice exhibit increased lethality compared to their wild-type (WT) counterparts 39. The *Dusp1*-deficient mice demonstrate elevated serum levels of CCL4, IL-10, and IL-6, accompanied by compromised spleen and liver function, as well as reduced bacterial clearance 39. Further underscoring the importance of Dusp1 in innate immunity, studies involving pneumonia mouse models infected with Chlamydophila pneumoniae reveal that *Dusp1^-/-^* mice elicit a heightened pro-inflammatory response, marked by an increase in cytokines (IL-6 and IL-1β) and chemokines (CCL3, CCL4, CXCL1, CXCL2) 40, contirbuting to abnormal pulmonary leukocyte infiltration. Complementing these findings, another investigation 41 underscores the protective role of Dusp1 in cardiac function, demonstrating that its overexpression in a TNF-α-induced septic cardiomyopathy mouse model preserves cardiomyocyte viability and reduces cardiac inflammation. Consistent with these results, our recent research indicates that Dusp1 downregulation is associated with a disruption in MQC, characterized by increased phosphorylation of Vcp in the context of endotoxemia-mediated myocardial injury 20. Thus, Dusp1 emerges as a pivotal regulator of the inflammatory response, potentially through multiple pathways, including but is not limited to inflammation, cytokine, chemokines and MQC.

A pivotal discovery of our study is the predominance of Dusp1 dephosphorylation as a molecular response to endotoxemia. In the milieu of LPS challenge, we detected a significant downregulation in the abundance of phosphorylated Dusp1. This alteration not only accelerates Dusp1 protein degradation in the cytoplasm but also highlights a crucial cellular response mechanism to endotoxemic stress. This observation aligns with previous studies. For instance, Choi *et al.* identified Dusp1 as a labile protein, prone to PKCδ-mediated degradation via the ubiquitin-proteasome pathway under glutamate exposure 42. Furthermore, it has been demonstrated that phosphorylation does not alter Dusp1's innate capacity to dephosphorylate members of the MAPK family. Instead, it stabilizes the Dusp1 protein itself, suggesting a regulatory mechanism wherein phosphorylation serves to enhance Dusp1 abundance within the cell 43. Additionally, analogous regulatory patterns are observed in other proteins such as Dusp16, where its dephosphorylation at Ser336 significantly extends its half-life and cytoplasmic retention 44. Given these findings, the phosphorylation status of Dusp1 is intricately linked to its protein stability and function. Recognizing the vital anti-inflammatory role of Dusp1 in inflammation-related pathologies, targeting its phosphorylation could represent an innovative strategy in the development of new anti-inflammatory therapeutics.

To unravel the upstream mechanisms responsible for Dusp1 dephosphorylation during endotoxemia, we employed co-IP coupled with mass spectrometry (MS) analysis of Dusp1. This led to the identification of Pgam1 as a critical phosphatase orchestrating Dusp1's post-transcriptional modification. Recently, Pgam1 has gained prominence as a versatile regulator in various inflammatory disorders. In a model of hepatocellular carcinoma (HCC), Pgam1 is reported to be a novel immunometabolic target and inhibition of Pgam1 promotes HCC ferroptosis through inducing CD8^+^ T-cell infiltration 45, suggesting a linkage between Pgam1 and inflammation. In fibroblasts, Pgam1 is significantly elevated by the hypoxic conditions and correlates with intracellular oxidative stress and inflammation damage 46. In the rabbit spinal cord, modulating Pgam1 expression is able to protect neuronal activity against isehcmia stress through preventing neuroinflammation 47. In addition to Pgam1, the other member of Pgam family, are also reported to be involved into the regulation of inflammation. The pathogenesis of LPS-caused acute lung injury has been associated with enhanced necroptosis and reduced mitochondrial fusion, consequent to Pgam5 activation 48. Inhibition of Pgam5 activation has been shown to protect mice from NKT cell-mediated acute liver damage by modulating mitochondrial integrity, underscoring the role of Pgam family-mediated mitochondrial dysfunction in immune signaling in hepatic tissue. This concept extends to inflammatory cell responses, where LPS-induced stress enhances the interaction between Pgam5 and Drp1, promoting macrophage polarization towards a proinflammatory phenotype and stimulating the production of inflammatory cytokines via NF-κB and MAPK pathway activation 49. In our study, we demonstrate that deletion of *Pgam1* can mitigate the inflammatory response in the heart following LPS exposure. Interestingly, the cardioprotective effect conferred by Pgam1 deficiency was significantly diminished by a Dusp1 dephosphorylation-mimicking mutant. These findings broaden our understanding of Pgam1's anti-inflammatory properties, highlighting Dusp1 dephosphorylation as a novel downstream event in response to Pgam1 upregulation. This insight expands the potential therapeutic scope of targeting the Pgam1/Dusp1 axis in inflammatory diseases.

The current study is encumbered by various constraints. Firstly, technological constraints precluded the generation of cardiomyocyte-specific Dusp1 overexpression mice. This limitation restricts our ability to isolate cardiac effects from systemic influences during endotoxemia. Furthermore, to establish the translational relevance of our findings, additional investigations using human samples are necessary. This step is crucial to validate the clinical significance of Dusp1 dephosphorylation and Pgam1 upregulation observed in our mouse models.

Overall, we delineate that LPS-induced endotoxemia-related myocardial dysfunction is characterized by a cascade of molecular events: upregulation of Pgam1, dephosphorylation of Dusp1, and a resultant disorder in MQC. The interaction between increased Pgam1 and Dusp1 fosters dephosphorylation and subsequent degradation of Dusp1, exacerbating the impairment in MQC and contributing to myocardial dysfunction in endotoxemia. Given these insights, targeting the Pgam1-Dusp1 axis emerges as a novel and promising therapeutic strategy. This approach holds potential for mitigating myocardial depression in patients suffering from endotoxemia, offering a new therapeutic avenue to address this complex cardiovascular complication.

## Supplementary Material

Supplementary figures and tables.

## Funding

National Natural Science Foundation of China (No. 82300315; No. 82374240; No. 82074369), Guangdong Province Basic and Applied Basic Research Fund Project (No. 2024A1515012174; No. 2024A1515013184). National Administration of Traditional Chinese Medicine Research Project (No. 0102023703), Project of the State Key Laboratory of Dampness Syndrome of Traditional Chinese Medicine jointly established by the province and the ministry (No. SZ2022KF10), Scientific Research Initiation Project of Guangdong Provincial Hospital of Traditional Chinese Medicine (No. 2021KT1709), Research Project of Guangdong Provincial Bureau of Traditional Chinese Medicine (No.20241120), Guangdong Provincial Key Laboratory of Research on Emergency in TCM (No. 2023B1212060062; 2023KT15450), Excellent Young Talents Program of Guangdong Provincial Hospital of Traditional Chinese Medicine (No. SZ2024QN05), Scientific Research Cultivation Project of Chinese Medicine Guangdong Laboratory (Grant No. HQL2024PZ036) and Basic Clinical Collaborative Innovation Program of Guangdong Provincial Hospital of Traditional Chinese Medicine and School of Biomedical Sciences, The Chinese University of Hong Kong (No. YN2024HK01).
