# Supplementary material for: Phosphoglycerate mutase 1-mediated dephosphorylation and degradation of Dusp1 disrupt mitochondrial quality control and exacerbate endotoxemia-induced myocardial dysfunction
Source: Theranostics. 2024 Nov 4;14(19):7488–504. doi: 10.7150/thno.102647 (PMC11626948; doi:10.7150/thno.102647)
Supplement: Supplementary file 1 — Supplementary figures and tables. [file thnov14p7488s1.pdf]

Supplemental Figures and Tables

Supplemental Table 1. Primers for qPCR

Gene	Forward Primer	Reverse Primer
Il-6	5'-CAGACTCGCGCCTCTAAGGAGT-3'	5'-GATAGCCGATCCGTCGAA-3'
Mcp1	5'-GATAGCCGATCCGTCGAA-3'	5′-GCTACCACAACATCTGGACATT-3′
Mmp9	5′-AACCAATGATGCTGGGTTCAC-3′	5'-GCGCCGACTCAGAGGTGT-3'
Gapdh	5′-TCGATATTGAGCGTCCAACCT-3′	5′-CAAAGGCACGTTTGGCATACA-3′
Dusp1	5′-CTCCTGGTTCAACGAGGCTATT-3′	5′-TGCCGGCCTGGCAAT-3′
Nrf2	5′-CCTCGCTGGAAAAAGAAGTG-3′	5′-GGAGAGGATGCTGCTGAAAG-3′
Pgc1α	5′-CGGAAATCATATCCAACCAG-3′	5′-TGAGGACCGCTAGCAAGTTTG-3′
Tfam	5′-GGCGAATTCCTCGAGGCCACCATG GCGCTGTTCCGGGGAATGT-3′	5′- CATACGCGTATGCTCAGAGATGTC TCCGGATCGT -3′

Supplemental Table 2. Antibody information

Name	Catalogue number	Dilution factor
GAPDH	Abcam, #ab8245	1:1000
Dusp1	Santa Cruz Biotechnology, # sc-373841	1:1000
$Dusp1^{S296}$	Bosterbio, #A02276S296	1:1000
$Dusp1^{S323}$	Amerigo, #A001485365STJ	1:1000
$Dusp1^{S364}$	St John's Laboratory, #SKU-STJ91170	1:1000
Parkin	Abcam, #ab77924	1:1000
Fundc1	Abcam, #ab224722	1:1000
LC3II	Abcam, #ab192890	1:1000
Beclin-1	Abcam, #ab207612	1:1000
Pgam1	Abcam, #ab288376	1:1000

Supplemental Figures

Supplemental Figure 1 *Dusp1* overexpression improves heart function. *Dusp1* transgenic (*$Dusp1^{Tg}$*) mice and its control literature *WT* mice were injected with lipopolysaccharide (LPS) at 10 mg/kg for 48 hrs to induce an endotoxemia myocardial model. Single cardiomyocytes were isolated from *$Dusp1^{Tg}$* mice and *WT* mice on a Langendorff apparatus and the mechanical properties of cardiomyocytes were measured. A. Echocardiography was used to determine cardiac function. **B.** Mechanical properties were measured in 100-120 cardiomyocytes per group. **C.** Survival data for *WT* mice and *$Dusp1^{Tg}$* mice. *p<0.05.

Supplemental Figure 1

A

Parameters	Vehicle *WT*	Vehicle *Dusp1^Tg*	LPS *WT*	LPS *Dusp1^Tg*
LVDd, mm	3.25±0.14	3.30±0.12	3.95±0.16*	3.56±0.14#
LVDs, mm	2.23±0.07	2.30±0.09	3.24±0.20*	2.53±0.11#
IVS, mm	0.81±0.03	0.82±0.03	0.69±0.05*	0.78±0.05#
PW, mm	0.82±0.05	0.81±0.06	0.71±0.06*	0.79±0.06#
FS, %	33.2±1.9	34.1±2.2	26.4±2.1*	31.6±1.5#
EF, %	63.5±4.4	62.2±4.3	43.6±5.7*	57.9±4.4#
E/A	1.29±0.21	1.32±0.17	0.84±0.09*	1.11±0.16#

LVDd, diastolic dimension of left ventricle; LVDs, systolic dimension of left ventricle; IVS, thickness of interventricular septum; FS, ratio of left ventricular fractional shortening; *p<0.05 vs. Vehicle+*WT* group, #p<0.05 vs. LPS+*WT* group.

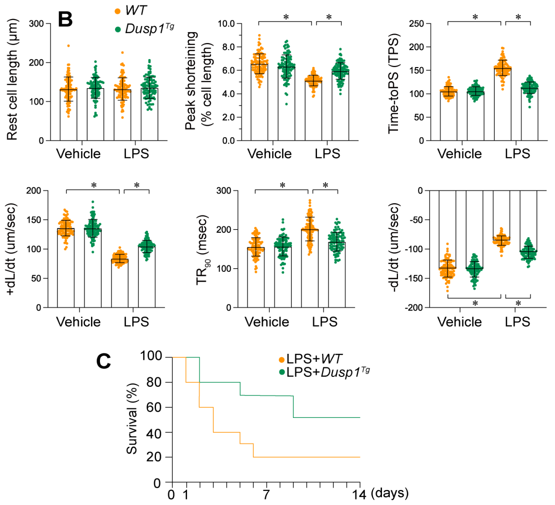

Supplemental Figure 2 *Dusp1* overexpression inhibits inflammation response and cardiomyocyte death. *Dusp1* transgenic (*Dusp1^Tg*) mice and its control literature *WT* mice were injected with lipopolysaccharide (LPS) at 10 mg/kg for 48 hrs to induce an endotoxemia myocardial model. A-C. RNA were isolated from heart tissues and the transcription of *Il-6*, *Mcp1*, and *Tnfα* were detected by qPCR. **D-E.** RNA were isolated from heart tissues and the transcription of *Icam1* and *Vcam1* were analyzed by qPCR. **F.** ELISA kit was used to detect the activity of caspase-3 in heart tissues. **G-I.** Serum were collected from mice after LPS exposure and the concentrations of BNP, TnT, and CK-MB were analyzed by ELISA. *p<0.05.

Supplemental Figure 2

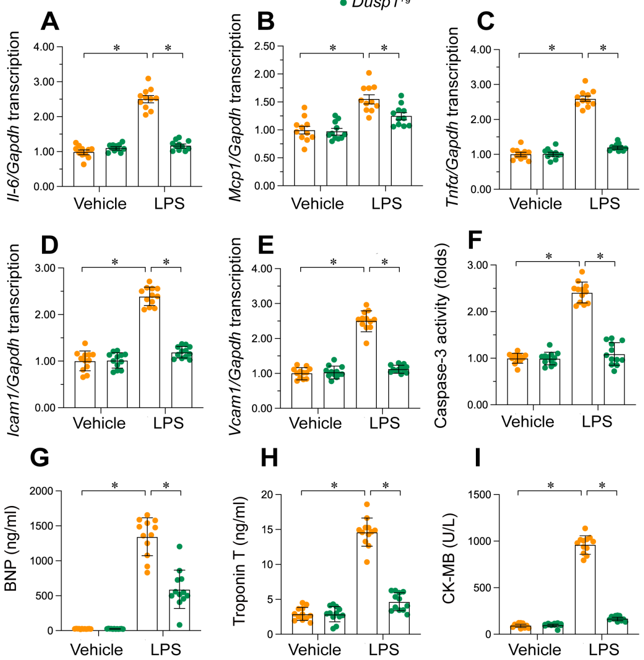

Supplemental Figure 3 Pharmacological inhibition of Pgam1 improved endotoxemia-mediated myocardial performance. WT mice were treated with PGMI-004A, an inhibitor of Pgam1, before lipopolysaccharide (LPS) injection at 10 mg/kg for 48 hrs to induce an endotoxemia myocardial

model. Single cardiomyocytes were isolated from mice on a Langendorff apparatus and the mechanical properties of cardiomyocytes were measured. A. Echocardiography was used to determine cardiac function. **B-G.** Mechanical properties were measured in 100-120 cardiomyocytes per group. **H-J.** RNA were isolated from heart tissues and the transcription of *Il-6, Mcp1,* and *Tnfα* were detected by qPCR. **K-M.** The concentration of serum TnT, CK-MB and BNP was determined by ELISA. *p<0.05.

Supplemental Figure 3

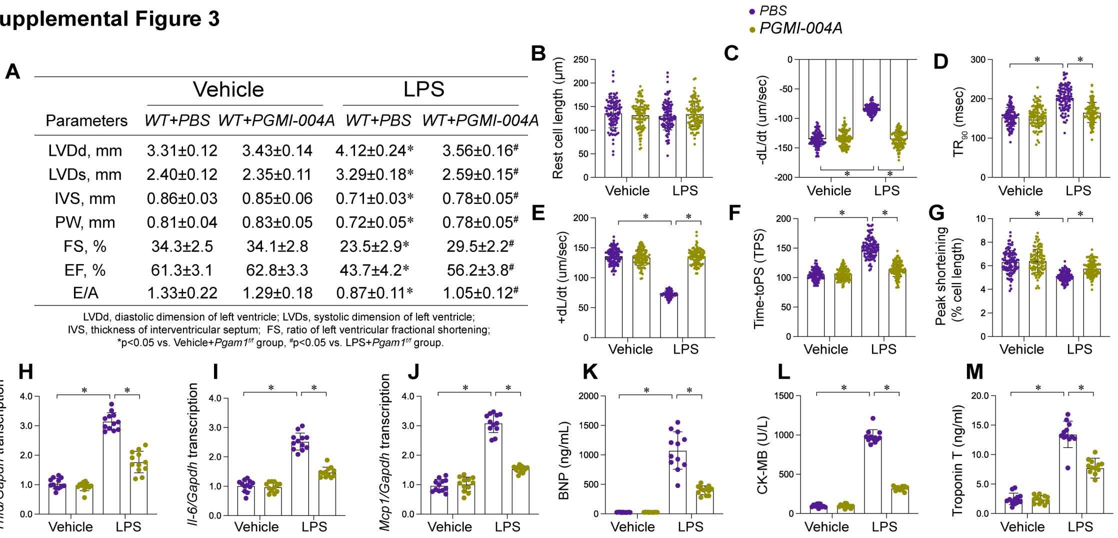

	Vehicle		LPS	
Parameters	*WT+PBS*	*WT+PGMI-004A*	*WT+PBS*	*WT+PGMI-004A*
LVDd, mm	3.31±0.12	3.43±0.14	4.12±0.24*	3.56±0.16#
LVDs, mm	2.40±0.12	2.35±0.11	3.29±0.18*	2.59±0.15#
IVS, mm	0.86±0.03	0.85±0.06	0.71±0.03*	0.78±0.05#
PW, mm	0.81±0.04	0.83±0.05	0.72±0.05*	0.78±0.05#
FS, %	34.3±2.5	34.1±2.8	23.5±2.9*	29.5±2.2#
EF, %	61.3±3.1	62.8±3.3	43.7±4.2*	56.2±3.8#
E/A	1.33±0.22	1.29±0.18	0.87±0.11*	1.05±0.12#

LVDd, diastolic dimension of left ventricle; LVDs, systolic dimension of left ventricle; IVS, thickness of interventricular septum; FS, ratio of left ventricular fractional shortening; *p<0.05 vs. Vehicle+*Pgam1*$^{f/f}$ group, #p<0.05 vs. LPS+*Pgam1*$^{f/f}$ group.
